# A novel approach for the endothelialization of xenogeneic decellularized vascular tissues by human cells utilizing surface modification and dynamic culture

**DOI:** 10.1038/s41598-022-26792-w

**Published:** 2022-12-24

**Authors:** Wen-Jin Ho, Mako Kobayashi, Kozue Murata, Yoshihide Hashimoto, Kenji Izumi, Tsuyoshi Kimura, Hideo Kanemitsu, Kazuhiro Yamazaki, Tadashi Ikeda, Kenji Minatoya, Akio Kishida, Hidetoshi Masumoto

**Affiliations:** 1 Department of Cardiovascular Surgery, Graduate School of Medicine, Kyoto University, 54 Shogoin Kawahara-cho, Sakyo-ku, Kyoto, 606-8507 Japan; 2 Department of Material-Based Medical Engineering, Institute of Biomaterials and Bioengineering, Tokyo Medical and Dental University, Tokyo, Japan; 3 Clinical Translational Research Program, RIKEN Center for Biosystems Dynamics Research, Kobe, Japan; 4 Institute for Advancement of Clinical and Translational Science, Kyoto University Hospital, Kyoto, Japan; 5 Tokai Hit., Co, Ltd., Fujinomiya, Japan; 6 Present Address: Department of Materials Processing, Graduate School of Engineering, Tohoku University, Sendai, Japan; 7 Present Address: Department of Cardiovascular Surgery, Kitano Hospital, Osaka, Japan

**Keywords:** Cardiology, Regenerative medicine, Tissue engineering

## Abstract

Decellularized xenogeneic vascular grafts can be used in revascularization surgeries. We have developed decellularization methods using high hydrostatic pressure (HHP), which preserves the extracellular structure. Here, we attempted ex vivo endothelialization of HHP-decellularized xenogeneic tissues using human endothelial cells (ECs) to prevent clot formation against human blood. Slices of porcine aortic endothelium were decellularized using HHP and coated with gelatin. Human umbilical vein ECs were directly seeded and cultured under dynamic flow or static conditions for 14 days. Dynamic flow cultures tend to demonstrate higher cell coverage. We then coated the tissues with the E8 fragment of human laminin-411 (hL411), which has high affinity for ECs, and found that Dynamic/hL411showed high area coverage, almost reaching 100% (Dynamic/Gelatin vs Dynamic/hL411; 58.7 ± 11.4 vs 97.5 ± 1.9%, *P* = 0.0017). Immunostaining revealed sufficient endothelial cell coverage as a single cell layer in Dynamic/hL411. A clot formation assay using human whole blood showed low clot formation in Dynamic/hL411, almost similar to that in the negative control, polytetrafluoroethylene. Surface modification of HHP-decellularized xenogeneic endothelial tissues combined with dynamic culture achieved sufficient ex vivo endothelialization along with prevention of clot formation, indicating their potential for clinical use as vascular grafts in the future.

## Introduction

Cardiovascular disease remains a major cause of death globally^1,2^. Several standardized options are available for treating ischemic heart and peripheral arterial diseases, such as vascular graft implantation^3^, stent implantation^4^, and drug intervention^5^. Among these, vascular graft implantation using autologous grafts provides high treatment efficacy, better quality of life, and prognosis^6^. Even though autologous vascular grafts are the most suitable for revascularization surgery targeting small arteries, graft preparation is not always ideal because of the poor quality of autologous grafts, possibly associated with comorbidity or previous surgery in the patient^7^, or shortage of preparation time owing to hemodynamic instability during surgery, especially in emergent cases. To address this dilemma, an advanced bioengineering graft platform needs to be established.

As a new paradigm to prepare vascular grafts as surrogate autologous grafts, decellularization of xenogeneic vascular tissue has recently been introduced, wherein immunological issues mediated by xenogeneic cells are mitigated^8,9^. Two major approaches are used for decellularization: chemical reagent-based^10^ and physical manipulation-based^11^ methods. To maintain the extracellular matrices (ECMs) after decellularization with a sufficient efficiency of decellularization and microorganism removal, we developed an original physical decellularization method called a high hydrostatic pressure (HHP) method^12–14^. We recently confirmed that implantation of xenogeneic HHP-decellularized vascular tissue in a large animal model could confer endothelial cell migration and maintenance of endothelium in the implanted vascular tissue^15^. However, thromboembolism occurred after implantation when the inner surface of the vascular grafts was damaged by dry conditions during surgery, possibly owing to the absence of an intact endothelial cell layer before implantation; this encouraged us to address this problem for the broader application of this technology.

Ex vivo recellularization is an approach used to reconstruct intact tissue structures, and can possibly help prevent acute thromboembolism after implantation^16^. To develop humanized vascular grafts from HHP-decellularized xenogeneic vascular tissues with biological vascular function, recellularization using human vascular cells might be an optimal approach. In particular, endothelialization of decellularized vascular tissues before in vivo implantation could be crucial to confer endothelial function to the graft, which might help prevent acute clot formation after implantation.

In the present study, we hypothesized and validated a novel method for the endothelialization of HHP-decellularized xenogeneic vascular tissues with human endothelial cells utilizing tissue surface modification by coating the tissue surface with an ECM that possesses high affinity with human endothelial cells, along with a dynamic flow culture method. We also confirmed that the optimized endothelialization protocol sufficiently prevented acute clot formation against human blood, and further validated this in an ex vivo test.

## Results

### The combination of coating with the E8 fragment of human laminin-411 and dynamic flow culture enables sufficient endothelial cell coverage on the decellularized vascular tissue

**Figure 1:**
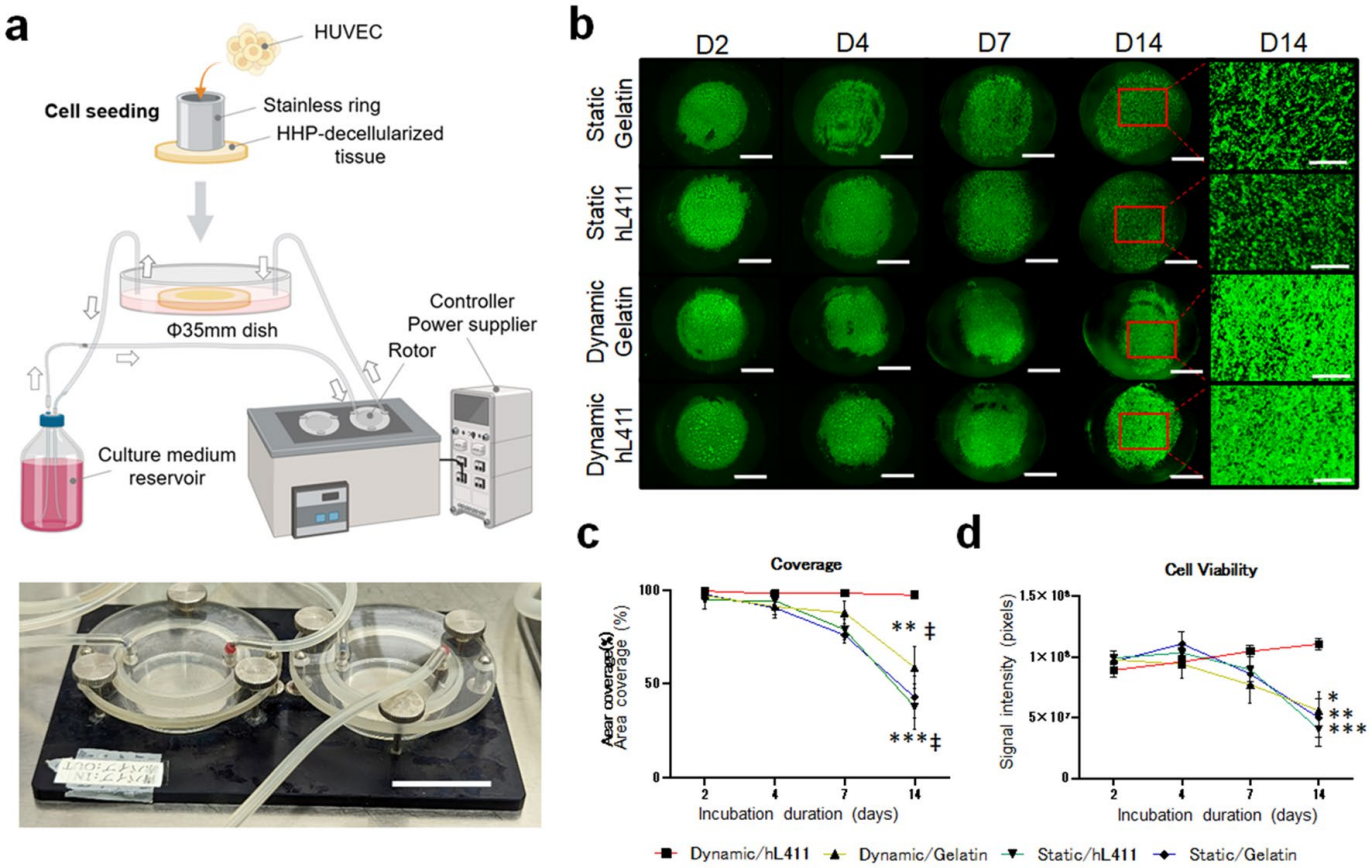
Cell viability assay. (**a**) A custom-made bioreactor system for dynamic cell culture. Upper: schematic of the entire system. Lower: the perfusion culture chamber. (**b**) Representative fluorescence images of cultured tissues. “D2” indicates “Two days of culture”. (**c**) Area coverage. (**d**) Signal intensity. HUVECs, human umbilical venous endothelial cells; HHP, high hydrostatic pressure; hL411, human laminin-411. Scale bars: 3 cm in (**a**), 5 mm in (**b**) (lower magnification) and 1 mm in (**b**) (higher magnification; rightmost column). *P < 0.05, **P < 0.01, ***P < 0.001 (vs Dynamic/hL411). †*P* < 0.05, ‡*P* < 0.01 (vs day 2).

Dynamic flow culture has been reported to improve the recellularization efficiency of decellularized pericardial tissues^17^, possibly owing to the reproduction of the physical conditions in the living body. We prepared a custom-made bioreactor that could confer dynamic flow of the culture medium and incubated gelatin-coated slices of porcine aorta endothelium seeded with human umbilical venous endothelial cells (HUVECs) under dynamic flow conditions (Fig. 1a). We found sufficient coverage of HUVECs on decellularized tissue in earlier incubation periods regardless of the dynamic condition (area coverage at day7: Static/Gelatin vs Dynamic/Gelatin; 76.0 ± 4.5 vs 88.0 ± 6.2%, *P* = 0.9881), but coverage started to decline after 7 days of incubation in the static incubation groups [Static/Gelatin: day2 vs day4 vs day7 vs day14; 97.8 ± 1.6 vs 90.7 ± 3.4 vs 76.0 ± 4.5 vs 42.8 ± 11.3%, *P* = 0.4342 (day2 vs day7), < 0.0001 (day2 vs day14), 0.0163 (day7 vs day14)]. Although the area coverage was 97.3 ± 1.4% at 2 days after incubation, coverage significantly declined at 14 days compared to that at 2 days even in dynamic culture [Dynamic/Gelatin: day2 vs day4 vs day7 vs day14; 97.3 ± 1.4 vs 91.3 ± 5.9 vs 88.0 ± 6.2 vs 58.7 ± 11.4%, *P* = 0.9992 (day2 vs day7), 0.0019 (day2 vs day14), 0.0631 (day7 vs day14)] (Fig. 1b,c).

Laminin-411 has a high affinity for human endothelial cells. To further improve the efficiency of endothelialization, we coated decellularized tissues with the recombinant E8 fragment of human laminin-411 (hL411)^18,19^. The combination of dynamic flow culture and coating with hL411 significantly improved the efficiency of coverage at 14 days after incubation, reaching almost 100% (area coverage at day14: Dynamic/Gelatin vs Dynamic/hL411; 58.7 ± 11.4 vs 97.5 ± 1.9%, *P* = 0.0017 (Fig. 1b,c). Further, the signal intensity of the covered area was significantly higher in the combination of dynamic culture and hL411 coating than that in the other groups [signal intensity at day14: Static/Gelatin vs Static/hL411 vs Dynamic/Gelatin vs Dynamic/hL411; 49.8 ± 16.0 vs 40.5 ± 14.0 vs 55.8 ± 15.0 vs 110.5 ± 4.7 pixels, *P* = 0.0027 (Static/Gelatin vs Dynamic/hL411), 0.0002 (Static/hL411 vs Dynamic/hL411), 0.0121 (Dynamic/Gelatin vs Dynamic/hL411)] (Fig. 1d). These results indicate that this combination might facilitate sufficient endothelialization with human endothelial cells on xenogeneic HHP-decellularized vascular tissues.

**Figure 2:**
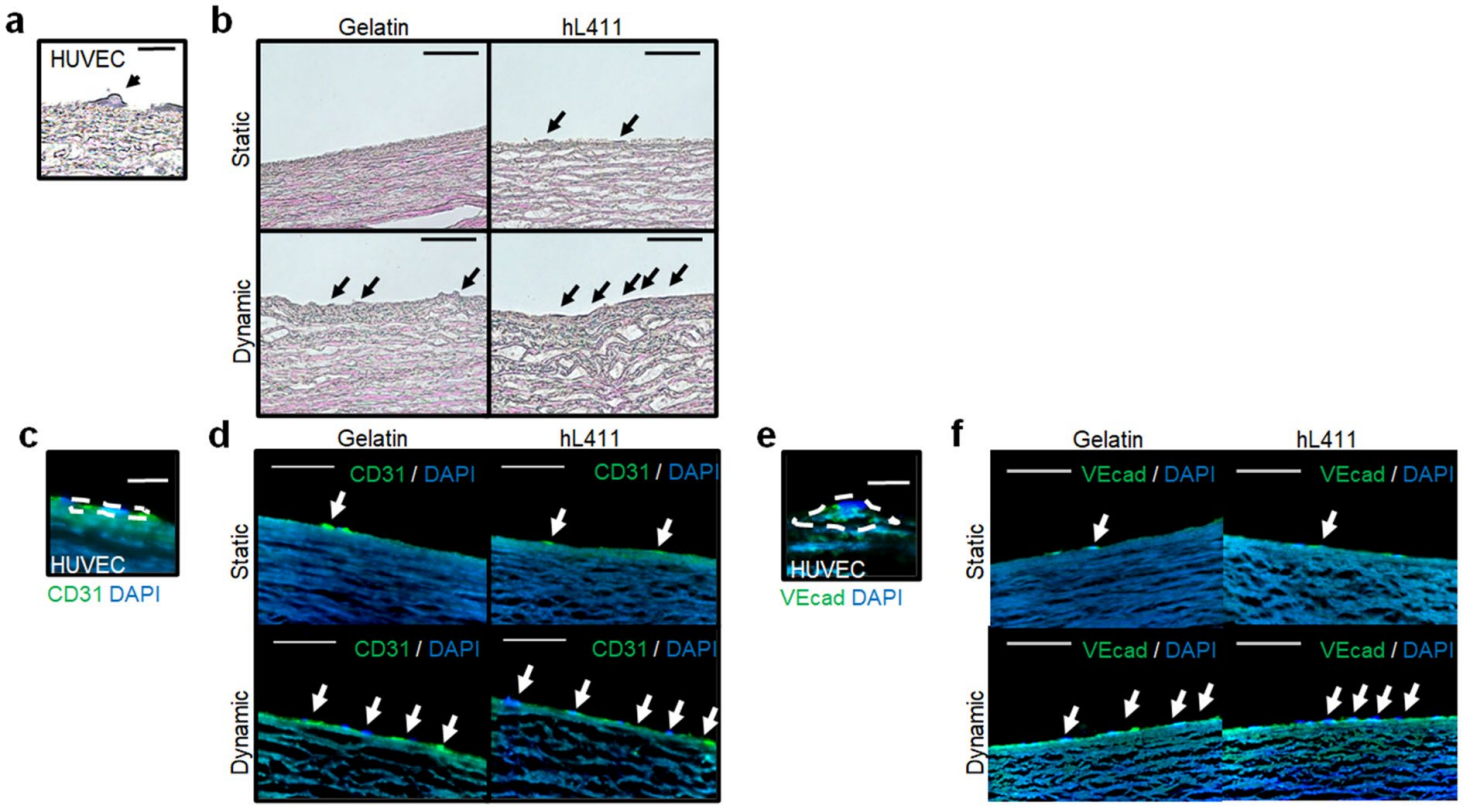
Histological evaluation of recellularized tissues. (**a**) Representative hematoxylin and eosin (H&E) staining for HUVECs (arrow). (**b**) Representative H&E staining for HUVECs in each culture condition. Arrows indicate HUVECs. (**c**) Representative immunohistochemical staining for CD31-positive HUVECs (dotted line). (**d**) Representative immunohistochemical staining for CD31-positive HUVECs in each culture condition. Arrows indicate HUVECs. (**e**) Representative immunohistochemical staining for VE-cadherin-positive HUVECs (dotted line). (**f**) Representative immunohistochemical staining for CD31-positive HUVECs in each culture condition. Arrows indicate HUVECs. DAPI, 4’,6-diamidino-2-phenylindole; VEcad, Vascular endothelial-cadherin. Scale bars: 10 µm in (**a**), (**c**) and (**e**); 100 µm in (**b**), (**d**) and (**f**).

To further confirm endothelialization, the recellularized vascular tissues were histologically evaluated. hematoxylin and eosin (H&E) staining revealed a thin, single layer of HUVECs on the tissue surface, especially in dynamic flow incubation, wherein which the cell layer had a higher amount and layer integrity than those in static incubation (Fig. 2a,b). Immunohistochemical staining for endothelial cells exhibited a higher extent of endothelial formation, similar to that in native endothelium comprising a single layer of endothelial cells, as an important morphological characteristic of the endothelium in combined culture conditions with dynamic culture and hL411 coating (Fig. 2c,d for CD31; Fig. 2e,f for Vascular endothelial-cadherin).

### Clot formation in human blood was reduced with the recellularized tissue prepared using the combination of dynamic flow culture and hL411 coating

**Figure 3:**
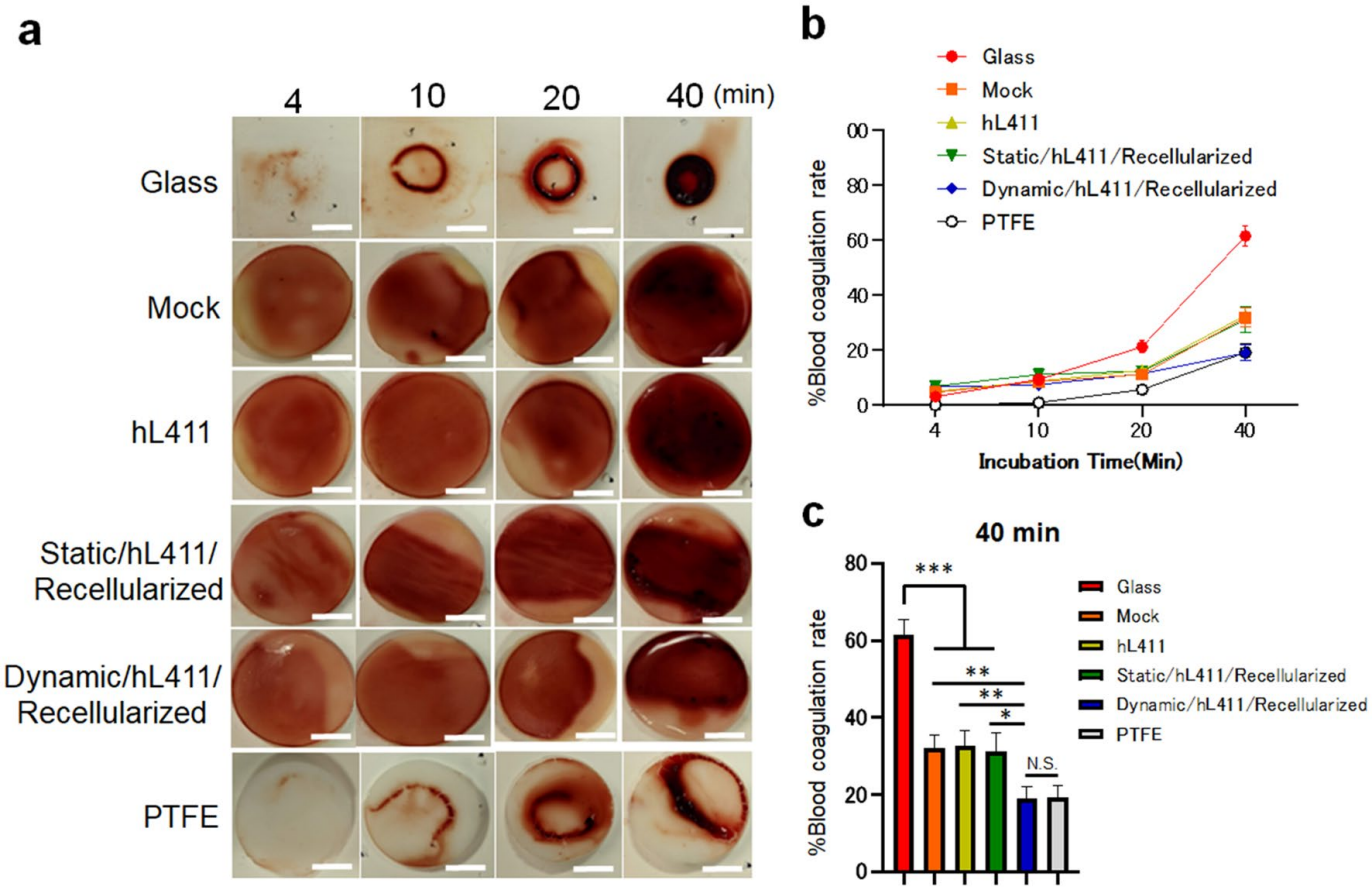
Ex vivo clot formation assay. (**a**) Representative images of samples at each time point after treatment with human blood. (**b**) %Blood coagulation rate. (**c**) %Blood coagulation rate at 40 min after treatment with human blood. PTFE, polytetrafluoroethylene. Scale bars: 5 mm. *P < 0.05, **P < 0.01, *P < 0.001, N.S., not significant.

We previously found that pretreatment of HHP-decellularized tissue suppresses clot formation when exposed to whole blood^20^. In the present study, we investigated whether endothelialization using human endothelial cells can mitigate clot formation in human whole blood (Fig. 3a,b). We thus conducted an ex vivo test to examine clot formation^20^ and found that the blood widely spread on the surface of the recellularized samples, whereas the blood did not spread and immediately clotted on glass (positive control of the clot formation assay). Samples prepared using combined dynamic incubation and hL411 coating resulted in significantly reduced clot formation compared to that obtained with the untreated group and was almost similar to that with a polytetrafluoroethylene (PTFE) sheet, which was used as a negative control [%Blood coagulation rate at 40 min: Glass vs Mock vs hL411 vs Static/hL411/Recellularized vs Dynamic/hL411/Recellularized vs PTFE; 61.6 ± 3.8 vs 32.0 ± 3.6 vs 32.7 ± 3.9 vs 31.2 ± 4.8 vs 19.0 ± 3.0 vs 19.2 ± 3.1%, *P* = 0.005 (Mock vs Dynamic/hL411/Recellularized, 0.0019 (hL411 vs Dynamic/hL411/Recellularized), 0.0121 (Static/hL411/Recellularized vs Dynamic/hL411/Recellularized), > 0.9999 (Dynamic/hL411/Recellularized vs PTFE)] (Fig. 3c). These results indicate that tissue endothelialization using the combined method in the present study can mitigate acute clot formation, which is an important biological function of the endothelium.

### Vessel container for the endothelialization of vascular-shaped tissues

**Figure 4:**
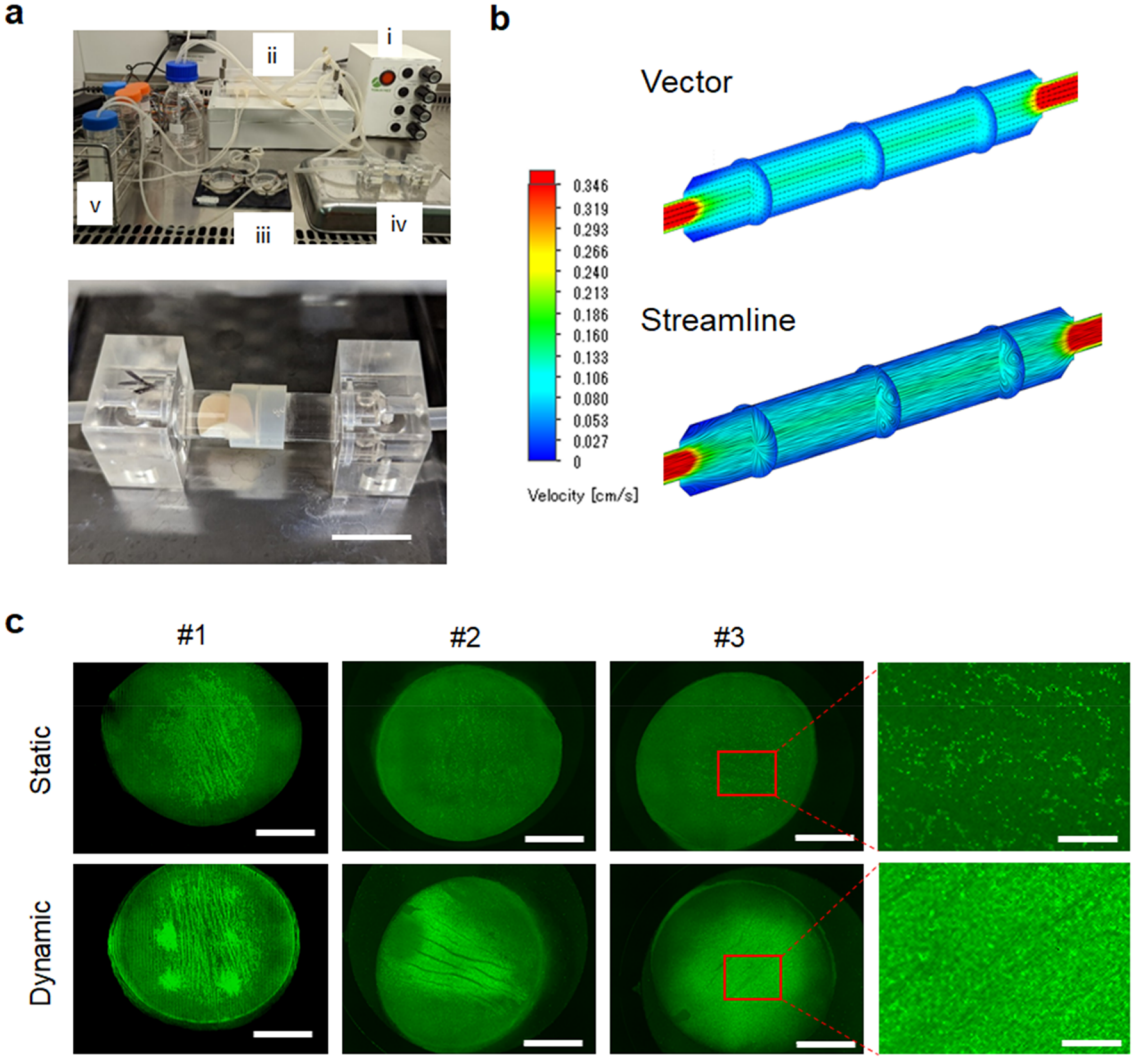
Recellularization of decellularized tissues using a vessel container. (**a**) A custom-made bioreactor system for dynamic cell culture using a vessel container. Upper: Entire system. (i) Console, (ii) Rotor, (iii) Plate type container (as shown in Fig. 1a), (iv) Vessel container, (v) Culture medium reservoir. Lower: Vessel container. Decellularized tissue is installed inside. (**b**) Flow simulation. Upper: Vector. Lower: Streamline. The vector image indicates the flow direction and travel in a tube container with arrow marks. The streamline image indicates the flow speed with colors. (**c**) Representative fluorescence images after static (upper) and dynamic (lower) culture. The results of three independent experiments are shown. Higher magnification images for #3 is also provided (right). Scale bars: 2 cm in (**a**) (lower), 5 mm in (**c**) (main) and 1 mm in (**c**) (right).

Endothelialization was first optimized with decellularized vascular tissues using thin slices of the porcine aorta as a plane structure. To investigate the application of this strategy in the vascular shape structure, we designed a tube-shaped vessel container in which we could insert the plane tissue and conduct dynamic flow incubation in a folded shape as in blood vessels (Fig. 4a; Supplementary Video 1). A computer-based flow simulation indicated a unidirectional regular fluid flow inside the vessel container without turbulence (Fig. 4b). After coating with hL411, we inserted the tissue into the container and conducted a dynamic flow incubation at 20 cm/s. After 14 days of incubation, we confirmed that the vessel container conferred an efficient level of recellularization similar to that in the plane tissue (Fig. 1a), even though a lacerated area without cells in the folded region was observed (Fig. 4c). These results indicate the possibility of sufficient endothelialization in vascular-shaped decellularized tissues using this vessel container, which can be then used in recellularization surgeries.

## Discussion

Decellularization has been investigated for a long time to develop new biomedical materials for regenerative medicine^21,22^. The advantages of decellularized tissues include a low immune response in xenografts and preserved tissue characteristics. A well-preserved tissue structure demonstrates structural strength and tolerance to high shearing force^23^. HHP-based decellularization is a procedure involving high hydrostatic pressure for cellular antigen annihilation, and is associated with less damage to the tissue structure^13,24,25^. Several reports have shown that ECM components may induce a host immune response, which may lead to the formation of blood clots even though the cellular antigens are removed by decellularization^26,27^. In the present study, we conducted endothelialization of decellularized tissue to reduce the direct exposure of ECM components, which resulted in suppression of clot formation, as confirmed by an ex vivo assay using human blood. However, we should recognize that we used frozen human blood after thawing instead of fresh blood which would be better for the recapitulation of clinical implantation of the vascular grafts. The capacity of less clot formation in our endothelialized tissues should be further validated through pre-clinical animal studies and subsequent clinical studies in our future work.

In the present study, we improved the efficiency of ex vivo recellularization over that using conventional methods by combining dynamic flow incubation and tissue surface modification. Our results indicate that this combination was necessary for sufficient recellularization of human endothelial cells as a single cell layer of the endothelium. Dynamic flow culture has been reported to improve the recellularization efficiency in various kinds of decellularized tissues^11,17,28^. In conventional methods of recellularization aiming for ex vivo tissue reconstruction before in vivo implantation, seeding cells by circulating a cell suspension (cells + culture medium) requires a relatively long duration of recellularization incubation, and results in a relatively low efficiency of recellularization^29,30^. We modified the protocol from previous reports to pre-seed the cells with a sufficiently high cell density and conducted dynamic flow incubation with cell-free medium for 2 weeks, which resulted in improved endothelialization. hL411 is known to exist at basement membrane of blood vessels and have a high affinity with integrin α6β1 expressing on the surface of ECs^31^. E8 fragment of hL411 is known as binding domain of integrin α6β1. Furthermore, hL411 promotes the differentiation of endothelial cells from human pluripotent stem cells^18,19^. The biological effect of hL411 coating on the homing of human endothelial cells might have further improved the endothelialization of decellularized tissue in this study.

The present approach has significant advantages for clinical applications. Our modified recellularization protocol provides endothelialized xenogeneic vascular tissues with human endothelial cells. Considering the abundant availability of xenogeneic vascular grafts from size-matched animals, a shortage of vascular grafts for revascularization surgery can be mitigated using the proposed methods. Human endothelial cells derived from human induced pluripotent stem cells (iPSCs) holding high affinity with hL411^18,19^ are a promising cell source^32^. Use of autologous, allogeneic human leukocyte antigen-matched human iPSCs from a healthy donor, obtained from an allogeneic iPSC bank or hypoimmunogenic iPSCs generated by gene editing might lead to less concerns regarding immunological problems after implantation^33,34^. Overall, mass production of recellularized vascular grafts mediated by sufficient hL411 coating and dynamic culture may confer a valuable medical resource, which is anticipated in revascularization surgeries for small arteries, which are increasing annually^35^. One the other hand, the possibility of tumor progression mediated by hL411 through excessive tissue angiogenesis should be noticed in clinical implementation of the present strategy^36^ and the optimal dose of hL411 should be further investigated.

In conclusion, we established sufficient endothelialization of HHP-decellularized vascular tissues using a combination of physical and biological approaches. These findings can contribute to the clinical use of small-caliber vascular bioengineered grafts that are suitable for revascularization surgeries in future.

## Methods

All methods were performed in accordance with the relevant guidelines and regulations (Declaration of Helsinki).

### HUVEC maintenance

The HUVEC line was purchased from PromoCell (Heidelberg, Germany) (#C-12200). Frozen HUVECs were thawed and cultured according to the manufacturer’s protocol. Upon confluency, the HUVECs were detached by 15 min incubation with trypsin–EDTA (0.25%) (Invitrogen, Waltham, MA, USA) and seeded onto culture dishes at a density of 500,000 cells/cm^2^ in Endothelial Cell Growth Medium 2 (Ready-to-use) (PromoCell, #C-22011) supplemented with Endothelial Cell Growth Medium 2 SupplementMix (PromoCell, #C-39216) (hereafter referred as “culture medium”). Cells from passages five to eight were used in the recellularization experiments. Cell morphology was microscopically checked, and cultures with abnormal cell shapes were excluded from further experiments.

### Preparation of decellularized porcine aorta slices

Decellularized porcine aorta slices were prepared as previously reported^20^. Briefly, fresh porcine aortas were purchased from Tokyo Shibaura Zouki, Tokyo, Japan and circularly cut with an inner diameter of 16 mm using a hollow punch after washing with saline. For decellularization, high hydrostatic pressure was applied at 1000 MPa and 30 °C for 10 min using a hydrostatic pressurization system (Dr. Chef, Kobelco, Tokyo, Japan); the samples were then washed with DNAse (0.2 mg/mL) and MgCl_2_ (50 mM) in saline at 4 °C for 7 days, followed by a change in the washing solution to 80% ethanol in saline at 4 °C for 3 days, and then to saline at 4 °C for 3 days to remove cell debris. The intima of the aorta was peeled off with tweezers and the resulting aortic slices were used for the experiment.

### Pre-treatment of decellularized tissue with hL411 and gelatin coating

To coat the aorta slices with hL411, we used iMatrix-411(#892041, Matrixome Inc., Suita, Japan), a recombinant E8 fragment of hL411. One microliter of the original titer was diluted in 200 µL of deionized water. The aorta slices were then coated with a 0.5 µg/cm^2^ of the diluted iMatrix-411 solution in accordance with the instruction of the manufacturer. To maintain a stable planar shape during the coating, the aorta slices were anchored using a stainless steel ring with 14 mm diameter (Fig. 1a). To check for fluid leakage, 200 µL of phosphate-buffered saline (PBS) was loaded into the ring. After completing the leakage check, PBS was discarded and the diluted iMatrix-411solution was added inside the stainless-steel ring. The outer region of the tissue was filled with PBS to maintain moist conditions. We incubated the tissue with the stainless ring in a 10 cm dish and placed the dish in a laminar flow cabinet at room temperature for approximately 2 h, in which the solution was expected to be almost vaporized. After the coating, the tissues were kept at 37 °C with 5% CO_2_ until the solution was fully vaporized but not completely dried. For gelatin coating, we used 0.1% gelatin from porcine skin (Sigma-Aldrich, St. Louis, MO, USA) diluted with distilled water. We covered the tissues with the gelatin solution for 15 min at room temperature and discarded the solution.

### Cell seeding onto the decellularized tissues

We placed the decellularized aorta slices in a 10-cm cell culture dish and anchored them using a stainless-steel ring (inner diameter 12 mm) to maintain the tissues in a planar shape. We then added 200 µL of PBS to the inner space of the stainless ring and kept it for 20 min at room temperature to check for fluid leakage. If PBS leakage was observed, the stainless-steel ring was repositioned. The PBS was discarded and cell suspension containing 2.0 × 10^6^ of HUVECs and 500 µL of culture medium was added followed by incubation at 37 °C with 5% CO_2_ for 24 h. After incubation, we removed the stainless-steel ring, replaced the culture medium, and incubated the tissues at 37 °C, with 5% CO_2_ for further 24 h.

### Dynamic flow culture

A custom dynamic flow culture system was prepared (Tokai Hit Co. Ltd., Fujinomiya, Japan). Briefly, it was assembled into three parts: a flow-generating rotor, an incubation container, and a rotor speed controller (Fig. 1a). The incubation container of the planar system was a stainless-steel platform with a thick glass cover having two input/output ports and was designed to fit a 35-mm cell culture dish. The vessel container for vascular-shaped tissues was made of polycarbonate; it was designed using SolidWorks 2019 software (Dassault Systems, Vélizy-Villacoublay, France) and fabricated using a milling machine (Fig. 4a). The container was designed in a separate format to facilitate tissue insertion. The inner diameter was 9 mm. The inner flow pattern (vector and streamline) was simulated using simulation software (SolidWorks Flow Simulation, Dassault Systems) (Fig. 4b).

To avoid bubble jamming in the incubation route, which may result in impaired cell culture, we rinsed the system with PBS prior to the culture medium. After washing with PBS, the fluid was replaced with the culture medium. After refilling the route with culture medium, we stopped the rotor, set the tissues installed in an incubation container, and restarted the dynamic flow culture. At the initial phase of the dynamic flow culture (1 h), we set the flow speed to 10% of the speed required for the maintenance phase (e.g., 10 rpm in 100 rpm of the maintenance rotor speed). After the initial phase, we increased the speed and maintained it at 200 rpm for planar tissue as well as for vascular-shaped tissue experiments. After confirming that the system was working without trouble, we placed the entire dynamic incubation system in a cell culture incubator (37 °C, 5% CO_2_) for further incubation. The incubation period was 2 weeks. The culture medium was changed every other day by refreshing approximately 10 mL of the culture medium in the culture medium reservoir. The rotor was stopped on day 14, and tissues were harvested for further examination.

### Cell viability assay

To evaluate cell viability, we used Calcein AM (Invitrogen) as an indicator of live cells^20^. We moved the tissue during incubation from the incubation container to a 12-well plate on days 2, 4, 7, and 14 for planar tissue experiments, and on day 14 for vascular-shaped tissue experiments, and then treated the tissues with Calcein AM solution diluted 1:1000 in endothelial cell growth medium. We then incubated the tissues for at least 35 min at 37 °C with 5% CO_2_. After incubation, the solution was discarded and the tissues 3 times with PBS. The fluorescence signal was measured using an all-in-one microscope (BZ-X800, Keyence, Osaka, Japan) and the attached software. Images were captured with a 5-layer overlay, where in each layer was 20 μm thick. The entire sample image was combined with 16 pieces of a single 2 × field (n = 6). Area coverage was calculated as the percentage of fluorescence-positive areas across the entire culture area. Signal intensity was calculated as the average signal intensity. We carefully conducted procedures to avoid drying of the tissues during evaluation.

### Histological evaluation

The recellularized tissues were collected and fixed overnight with 4% paraformaldehyde (PFA), followed by embedding in OCT compound (Sakura Finetek Japan, Tokyo, Japan) and freezing for further cryosectioning. Sections of 6 μm thickness were prepared and stained with hematoxylin and eosin. For immunofluorescence staining for CD31, sections were treated with Protein Block Serum Free (DAKO, Glostrup, Denmark) and incubated overnight at 4 °C with a primary antibody against human endothelial cells; rabbit polyclonal anti-CD31 antibody, ab28364 (Abcam, Cambridge, UK) (1:150). Then, anti-rabbit Alexa Fluor 488 (1:2000) was used as the secondary antibody, and the sections were incubated for 60 min at room temperature. For Vascular endothelial-cadherin, we used a fluorescein isothiocyanate (FITC)-conjugated antibody (FITC Mouse anti-Human CD144, Clone 55-7H1, BD Biosciences, Franklin Lakes, NJ, USA) (1:100) without secondary antibody. Cell nuclei were visualized using 4',6-diamidino-2-phenylindole (DAPI) staining (0.1 µg/ml). All sections were photographed using an all-in-one microscope (BZ-X800; Keyence). The evaluations were repeated three times.

### Clot formation assay

A clot formation assay was conducted as described in our previous report, with some modifications^20^. Frozen human single donor whole blood was purchased (Human Heparin sodium whole blood, single donor, CTSAG050, BioIVT, Westbury, NY, USA) and aliquoted into small volumes (2 mL/tube), stocked in − 20 °C freezer and pre-warmed using a 37 °C water bath before use. For activating the blood sample, 0.343% citric acid and 0.004 M CaCl_2_ was used. The samples obtained after 14 d of incubation (n = 6) were placed on a wet paper towel floating in a 37 °C water bath. We gently mixed activated whole blood and dropped 50 µL of blood for each sample. Samples were harvested at 4, 10, 20, and 40 min and then gently washed with PBS. The samples were then placed on white paper to observe the extent of clot formation (Fig. 4a). We then prepared 2 mL of deionized water in a 12-multiwell plate, immersed the samples with clots, and stored the samples overnight at 4 °C. We collected 1 mL of the supernatant, centrifuged it for 15 min at 1500 × *g*, 22 °C. The supernatant was added to a 96-multiwell plate (200 µL/well). The amount of dissolved hemoglobin was measured using a microplate reader at a wavelength of 540 nm (iMark, Bio-Rad, Hercules, CA, USA). %Blood coagulation rate was calculated as follows: (Sample read value—Blank read value)/(Positive control read value—Blank read value) × 100 (%). Glass coverslips were used as positive controls, mock samples (decellularized tissue without recellularization) were used as internal controls, and polytetrafluoroethylene (PTFE) sheets were used as negative controls. For the microplate reading, we used 50 µL of whole blood in 2 mL of deionized water as a positive control and deionized water as a negative control. This experiment was repeated six times.

### Statistical analysis

Data were processed using GraphPad Prism 8 software version 8.3.0. (San Diego, CA, USA). Values are expressed as mean ± SD. Statistical analysis was performed using two-way repeated-measures analysis of variance with Tukey’s post-hoc test. Statistical significance was set at *P* < 0.05.

## Supplementary Information

Supplementary Legends.

Supplementary Video 1.

## Data Availability

All data generated or analyzed during this study are available in accordance with reasonable requests to the corresponding author.
