# Supplementary material for: A novel approach for the endothelialization of xenogeneic decellularized vascular tissues by human cells utilizing surface modification and dynamic culture
Source: Sci Rep. 2022 Dec 24;12:22294. doi: 10.1038/s41598-022-26792-w (PMC9789980; doi:10.1038/s41598-022-26792-w)
Supplement: Supplementary file 1 — Supplementary Legends. [file 41598_2022_26792_MOESM1_ESM.docx]

**Supplementary video legend**

**Supplementary video 1: Procedure of the installation of decellularized tissues into the vascular-shape container.**

The video of the procedure is provided.
